# Development and Characterization of Electrospun Composites Built on Polycaprolactone and Cerium-Containing Phases

**DOI:** 10.3390/ijms241814201

**Published:** 2023-09-17

**Authors:** Cristiana Plocon, Alexandru Evanghelidis, Monica Enculescu, Gabriela Isopencu, Ovidiu Oprea, Mihaela Bacalum, Mina Raileanu, Sorin Jinga, Cristina Busuioc

**Affiliations:** 1 University Politehnica of Bucharest, RO-060042 Bucharest, Romania; ploconcristiana@gmail.com (C.P.); gabriela.isopencu@gmail.com (G.I.); ovidiu.oprea@upb.ro (O.O.); sorinionjinga@yahoo.com (S.J.); 2 National Institute for Materials Physics, RO-077125 Magurele, Romania; alex.evanghelidis@infim.ro (A.E.); mdatcu@infim.ro (M.E.); 3 National Institute of Physics and Nuclear Engineering, RO-077125 Magurele, Romania; mihaela.bacalum@nipne.ro (M.B.); mina.raileanu@nipne.ro (M.R.)

**Keywords:** CeO_2_, calcium phosphates, bioglass, polycaprolactone, electrospinning, scaffolds

## Abstract

The current study reports on the fabrication of composite scaffolds based on polycaprolactone (PCL) and cerium (Ce)-containing powders, followed by their characterization from compositional, structural, morphological, optical and biological points of view. First, CeO_2_, Ce-doped calcium phosphates and Ce-substituted bioglass were synthesized by wet-chemistry methods (precipitation/coprecipitation and sol-gel) and subsequently loaded on PCL fibres processed by electrospinning. The powders were proven to be nanometric or micrometric, while the investigation of their phase composition showed that Ce was present as a dopant within the crystal lattice of the obtained calcium phosphates or as crystalline domains inside the glassy matrix. The best bioactivity was attained in the case of Ce-containing bioglass, while the most pronounced antibacterial effect was visible for Ce-doped calcium phosphates calcined at a lower temperature. The scaffolds were composed of either dimensionally homogeneous fibres or mixtures of fibres with a wide size distribution and beads of different shapes. In most cases, the increase in polymer concentration in the precursor solution ensured the achievement of more ordered fibre mats. The immersion in SBF for 28 days triggered an incipient degradation of PCL, evidenced mostly through cracks and gaps. In terms of biological properties, the composite scaffolds displayed a very good biocompatibility when tested with human osteoblast cells, with a superior response for the samples consisting of the polymer and Ce-doped calcium phosphates.

## 1. Introduction

In tissue engineering, biomaterials play an essential role by reproducing the mechanical and biological functions of the extracellular matrix in body tissues, working as an artificial extracellular matrix [1,2]. These biomaterials act like a *3D* porous support for cells to penetrate and produce new tissues with appropriate structures and functions [3]. The key properties include biocompatibility, biodegradability and bioresorbability to allow tissue replacement without unwanted effects, such as inflammation [4].

Among the most notable technological advances in tissue engineering are the resorbable porous scaffolds [2,5]. The optimized versions can be fabricated only by precise control of their properties, such as pore shape, diameter, orientation and volume fraction, but also matrix chemical composition and microstructure [6]. Moreover, the transition from biostable to bioresorbable implants represented a significant evolutionary step in biomaterials research [7].

Polymers are the most used biomaterials for the manufacture of scaffolds [8]. Due to their mechanical properties and degradation rates that match those of soft and hard tissues, they are ideal candidates for the development of synthetic bone and vascular grafts [9]. Polycaprolactone (PCL) is a semicrystalline linear polymer that is flexible and hydrophobic, as well as easy to process [8]. It has been integrated in a wide range of medical applications, addressing wound healing [10], drug delivery [11] and soft [12] or hard [13] tissue engineering. PCL degrades through a hydrolytic mechanism under physiological conditions, with the resulting fragments at a small molecular level being captured by macrophages and reduced intracellularly [14]. Moreover, the slow degradation rate (total degradation in about 2–4 years, depending on quantity) allows the corresponding scaffold to remain intact long enough to allow new tissue formation and, in the end, its complete replacement, so that no subsequent surgical intervention will be required for the implantable device extraction [15]. At the same time, the scaffold must provide sufficient mechanical support during the degradation process to ensure the integrity and temporary functionality of the regenerating tissue.

Electrospinning is a widely used technology for electrostatically obtaining fibres with diameters ranging from 2 nm to several micrometres from polymer solutions of both natural and synthetic polymers [16]. Lately, it has become one of the most widely used fabrication methods for the preparation of nanofibrous scaffolds with applications in tissue engineering [17]. Typical synthetic polymers used in the biomedical field are hydrophobic biodegradable polyesters, among which PCL [18], polylactic acid (PLA) [19] and polyglycolic acid (PGA) [20] can be listed. PCL has frequently been combined with mineral bioactive phases, the most approached one being hydroxyapatite [21,22], a strategy that has led to multifunctional materials with improved potential in bone regeneration.

On another hand, dopants are often employed in tissue engineering to enhance the mechanical, biochemical and biological properties of different parent matrices. From the large variety of dopants, rare earth (RE) elements have attracted increased attention due to their special characteristics, playing an important role in refining scaffold performance [23]. Their cations possess unique biological features required for effective bone regeneration, such as osteogenic, angiogenic, antimicrobial and immunomodulatory activities [24]. Cerium (Ce) [25], europium (Eu) [26] and samarium (Sm) [27] are just three examples of elements with demonstrated potential in developing medical applications.

Cerium oxide (CeO_2_) nanoparticles represent important antibacterial agents due to their low toxicity to normal cells and distinct mechanism of antibacterial action, which is based on the reversible conversion between the two valence states of Ce (3+ and 4+), subsequently associated with the formation and migration of oxygen vacancies [28]. The interaction between CeO_2_ nanoparticles and the bacterial membrane is the crucial step in determining their toxicity. After CeO_2_ nanoparticles are adsorbed on bacterial membranes, based on electrostatic attraction, they can affect bacterial viability through mechanical effects, inducing oxidative stress or interfering with nutrient transport functions [29,30]. The dual characteristics of CeO_2_ nanoparticles, which can act as natural antioxidants and protect cells from oxidative stress, but can also exhibit toxicity under certain conditions, give them multiple applications in the field of biomedicine [31,32]. However, the toxic performance on different bacterial colonies depends on various factors, including synthesis methods and operating conditions [33]. In this context, the precipitation route is a simple method by which a precipitate is separated through chemical reactions from a primary solution containing the targeted ions [28], while the sol-gel approach is a complex chemical technique involving the transformation of the precursor solution into a *3D* gel by controlled hydrolysis and condensation of the precursors [34]. Moreover, it was reported that nanoceria-containing polymeric composites combine the advantages exhibited by each component and reduce the intrinsic drawbacks of both nanoceria and polymeric phases [35].

When used as a dopant, Ce can be integrated in different types of host matrices, both vitreous and crystalline, targeting the achievement of additional valuable biological properties, such as bioactivity. Calcium phosphates [36,37] and bioglasses [38,39] are two of the most investigated systems that have multiple doping ions and high doping concentrations. Additionally, the combination of such bioactive phases with bioresorbable polymers has also been extensively studied as innovative architectures with targeted multifunctionalities [40,41].

Atkinson et al. [42] synthesized Ce-containing mesoporous bioactive glasses by an evaporation-induced self-assembly method and concluded that the increase in ceria content up to 5 mol% improves the chemical stability without significantly altering bioactivity. Zheng et al. [43] developed Ce-doped mesoporous bioactive glass nanoparticles by a post-impregnation strategy and highlighted their great potential in treating bone defects under inflammatory conditions, considering their antioxidant, anti-inflammatory and pro-osteogenesis activities. Leu Alexa et al. [44] printed composite materials based on gelatine methacryloyl and hydroxyapatite doped with Ce ions and showed their capacity for osteogenic differentiation. Sousa et al. [45] precipitated Ce-doped calcium phosphates on bacterial cellulose as a platform for controlling the *3D* architecture, followed by thermal treatment at 600 °C, which induced a trabecular morphology similar to bone. Fernandes et al. [46] produced PCL membranes incorporated with 58S bioactive glass doped with Zn by electrospinning and reported increased hydrophilicity, as well as suitable conditions for cell activity and differentiation.

Considering all the above information, the design and development of composite materials with complex composition and controlled morphological properties seems to be a promising strategy for achieving unique or personalized implantable devices. Thus, by combining a bioresorbable polymer with a bioactive phase and an antibacterial agent, a new type of scaffold that responds to most of current medical requirements could be proposed. Therefore, in this work, PCL electrospun scaffolds were loaded with Ce-containing mineral powders, such as CeO_2_, Ce-doped calcium phosphates and Ce-substituted bioglass. In this way, the polymer bioresorbability was supplemented with phosphates, bioglass bioactivity and Ce antimicrobial activity, leading to multifunctional composite materials for bone engineering. To our knowledge, such ternary systems were not reported until now, opening the perspective to a novel generation of hard-tissue substitutes.

## 2. Results and Discussion

### 2.1. Characterization of Powders

**Figure 1:**
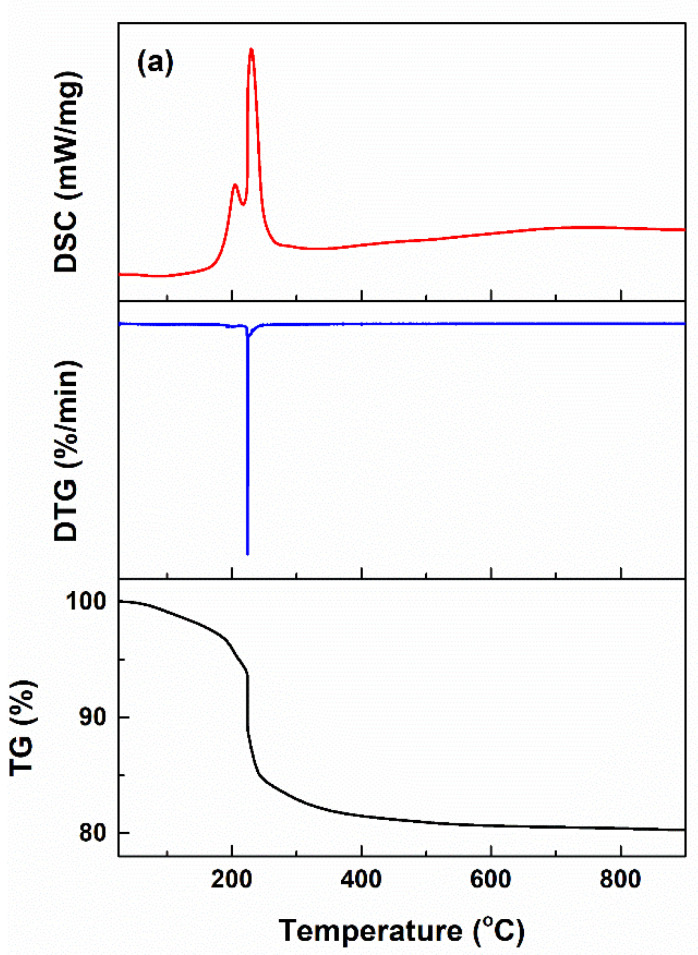

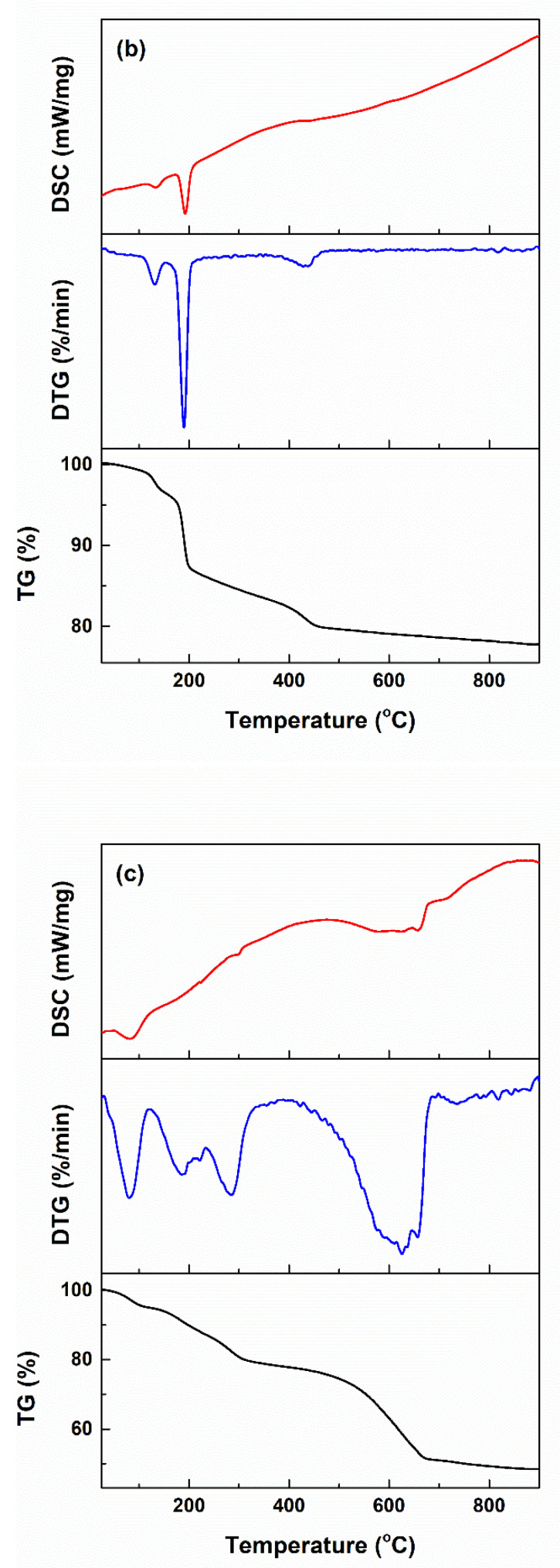
Complex thermal analysis of (**a**) CeO_2_, (**b**) CP-Ce dried precipitates and (**c**) BG-Ce dried gel. TG—thermogravimetric curve, DTG—derivative of the thermogravimetric curve in relation to time, DSC—differential scanning calorimetry.

The dried precipitates and gel were subjected to thermal analysis up to 900 °C to identify the appropriate calcination temperatures (Figure 1). Figure 1a displays the resulting curves in the case of the CeO_2_ intermediate, for which a single main step of weight loss of approximately 9.3%, centred at 224 °C, can be observed. According to the DSC curve, no endothermic effect assigned to Ce(OH)_4_ dehydroxylation can be identified, but a complex asymmetric exothermic effect is present in the range of 150–280 °C. The latter can be attributed to CeO_2_ crystallization, which occurs concomitantly with the dehydroxylation process, this being integrated in the final effect. The total mass loss from room temperature to 900 °C is around 19.7%, while up to 500 °C is around 19.1%, meaning that the application of a calcination treatment at 500 °C will ensure the removal of most residues and chemically bound water, together with CeO_2_ crystallization.

The thermal analysis performed on the CP-Ce intermediate is shown in Figure 1b. There are three main stages of weight loss, associated with three endothermic processes. The first one occurs in the range of 110–150 °C (~2.5%) and represents the evaporation of residual or adsorbed water; the second takes place in the range of 160–210 °C (~9.4%) and is attributed to the removal of chemically bound water; and the third one take place in the range of 390–470 °C (~2.8%) and highlights the decomposition of residual nitrate. Thus, the total mass loss from room temperature to 900 °C is around 22.2%, while up to 800 °C is around 21.8%, which means that most of the gas-generating compounds are eliminated up to 800 °C. However, 500 °C was also selected as the calcination temperature, since the weight loss was around 20.4%: more than 90% of unwanted compounds were removed.

Figure 1c presents the thermal analysis for the BG-Ce intermediate, where at least four main stages of weight loss, all accompanied by endothermic effects, can be seen. These have the following characteristics: 30–120 °C (~5.0%), 120–235 °C (~7.9%), 235–330 °C (~7.8%) and 430–680 °C (~25.9%). They are assigned to the following processes: volatilization of residual solvents and adsorbed water, removal of chemically bound water, preliminary decomposition of residual nitrates and completion of nitrate decomposition. WE concluded that the total mass loss from room temperature to 900 °C is around 51.5%, while up to 800 °C is around 50.7%, which recommends this temperature for calcination.

**Figure 2:**
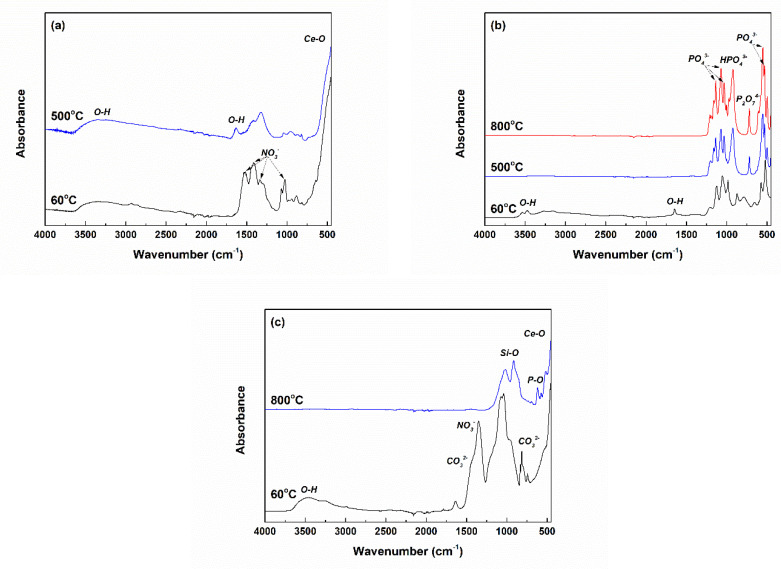
ATR-FTIR spectra of the dried precursors (precipitates and gel) and calcined powders: (**a**) CeO_2_, (**b**) CP-Ce and (**c**) BG-Ce.

The ATR-FTIR spectra recorded on both as-prepared and calcined powders are centralized in Figure 2. In the case of CeO_2_ (Figure 2a), the presence of NO_3_^−^ groups coming from the Ce precursor is confirmed through the vibrational bands that emerged in the range of 1000–1600 cm^−1^. The calcination process performed at 500 °C led to the disappearance of these bands, demonstrating the removal of all residues. Below 700 cm^−1^, an intense vibrational band corresponding to Ce–O bonds can be observed [47]. The other small contributions profiled between 800 and 1600 cm^−1^ seem to be the fingerprint of CeO_2_ as well [48]. Moreover, the absorbed water molecules give two representative vibrational bands at 1630 and 3400 cm^−1^.

Regarding CP-Ce (Figure 2b), the ATR-FTIR spectra indicate PO_4_^3−^ (532, 550, 1030, 1070 and 1135 cm^−1^), HPO_4_^3−^ (923 cm^−1^) and P_2_O_7_^4−^ (720 cm^−1^), respectively [49].

When it comes to BG-Ce (Figure 2c), the vibrational bands placed in the range of 800–1200 cm^−1^ are assigned to Si–O bonds and the band centred at 620 cm^−1^ to P–O bonds, while at lower wavenumber values the contributions of Ca–O and Ce–O bonds are found [50]. The residual groups (OH^−^ and NO_3_^−^) or accidentally attached entities (OH^−^ and CO_3_^2−^), evident in the dried gel, are no longer present in the calcined powder [50].

**Figure 3:**
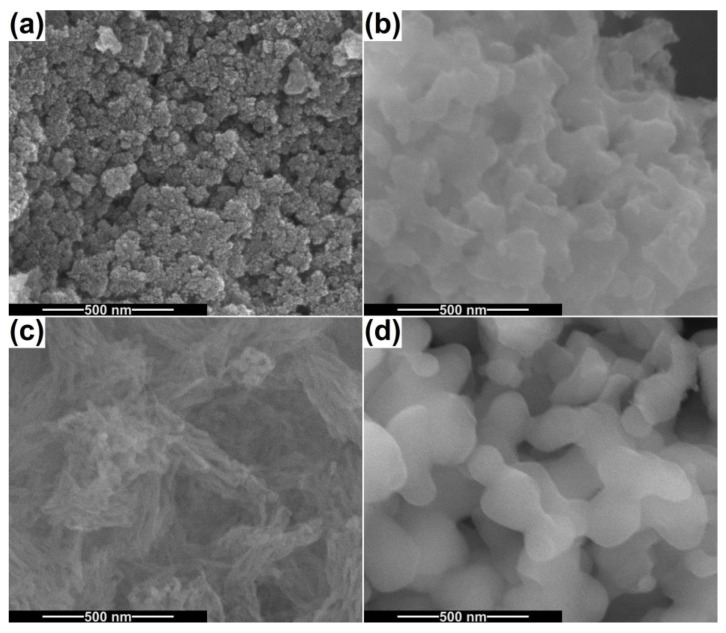
SEM images of the calcined powders: (**a**) CeO_2_, (**b**) BG-Ce, (**c**) CP-Ce-5 and (**d**) CP-Ce-8.

Figure 3 shows SEM images recorded for the powders calcined at 500 and 800 °C. The CeO_2_ sample consists of bunches of about 100 nm in size, made up of quasi-spherical particles with dimensions below 10 nm (Figure 3a). Their size distribution is monomodal and extremely narrow, confirming the suitability of the precipitation method for synthesizing nanoparticles with controlled diameters. The second powder prepared through coprecipitation is CP-Ce, in its variants CP-Ce-5 and CP-Ce-8, and it exhibits a completely different morphology. The first one is loose, with elongated fine particles, almost acicular, whose size cannot be measured with precision but are connected in a porous and fragile network (Figure 3c). The second one is well defined, with rounded and coarse particles up to 100 nm in dimension, forming a *3D* scaffold due to a high number of bridges that emerged between neighbouring particles (Figure 3d). The BG-Ce sample contains rugged particles trapped in a continuous matrix crossed by channels, with the individual blocks being micrometric (Figure 3b).

**Figure 4:**
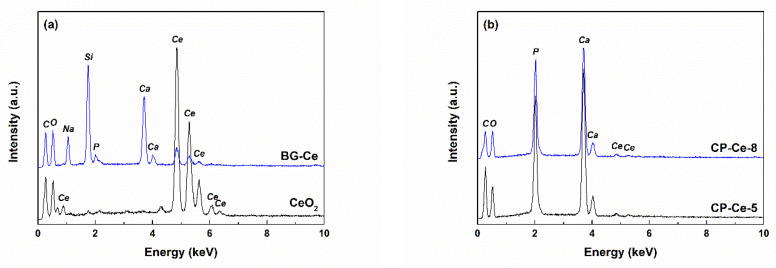
EDX spectra of the calcined powders: (**a**) CeO_2_, BG-Ce and (**b**) CP-Ce.

The elemental composition was studied by EDX spectroscopy, and the spectra registered on the calcined specimens are available in Figure 4. The CeO_2_ powder includes Ce and O, but C is also present due to sample fixation on carbon tape. In addition to Ce and O, more spectral lines can be observed in the case of the BG-Ce powder, such as Si, P, Ca and Na, in correlation with the designed composition. The difference in intensity for Ce peaks is understandable since the concentration of Ce in CeO_2_ is much higher compared to its concentration in bioglass, where it was integrated as substituent at a proportion of 5 mol% (Figure 4a). The CP-Ce powders have a composition based on Ca, P and O, to which Ce is added, but low in content as the shallow specific peaks suggest, a fact that is not surprising considering its role as a dopant.

**Figure 5:**
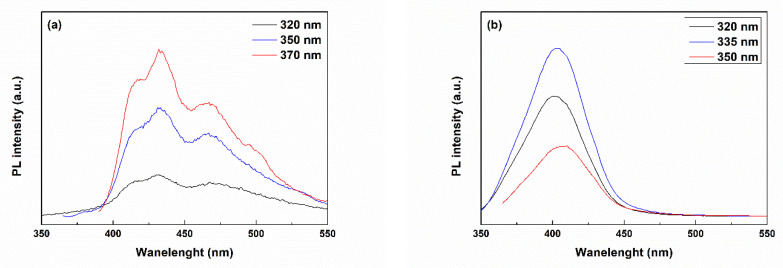
PL spectra of the calcined powders: (**a**) CeO_2_ and (**b**) BG-Ce.

The PL spectra recorded at room temperature and employing different excitation wavelengths are displayed in Figure 5. A large emission band can be seen between 390 and 520 nm for the CeO_2_ sample, suggesting that the surface defects are prevailing. All PL spectra obtained after excitation at various wavelengths show a strong maximum at 432 nm and three weaker maxima around 417, 467 and 495 nm. This broad complex band can be explained by the fact that different shapes and sizes of particles can influence the excited charge carriers and then their energy relaxation on the crystal surface, leading to variable photoluminescence properties [51]. In other words, if the first peak could be attributed to the band-to-band transition (from the conduction band to the valence band), the surface defects are responsible for the other emissions, namely electrons hopping from different defect levels to the valence band. There are multiple defect-related contributions because oxygen vacancies can trap electrons (one or two) or not [52,53]. Moreover, for the creation of each oxygen vacancy in CeO_2_, two neighbouring Ce^4+^ ions are reduced to Ce^3+^ ions [54].

As far as CP-Ce is concerned, the PL analysis did not provide significant information, probably in correlation with Ce integration in the crystal lattice of calcium phosphates or a lower degree of precipitation for Ce^4+^ when in solution with Ca^2+^ and PO_4_^3−^ ions.

**Figure 6:**
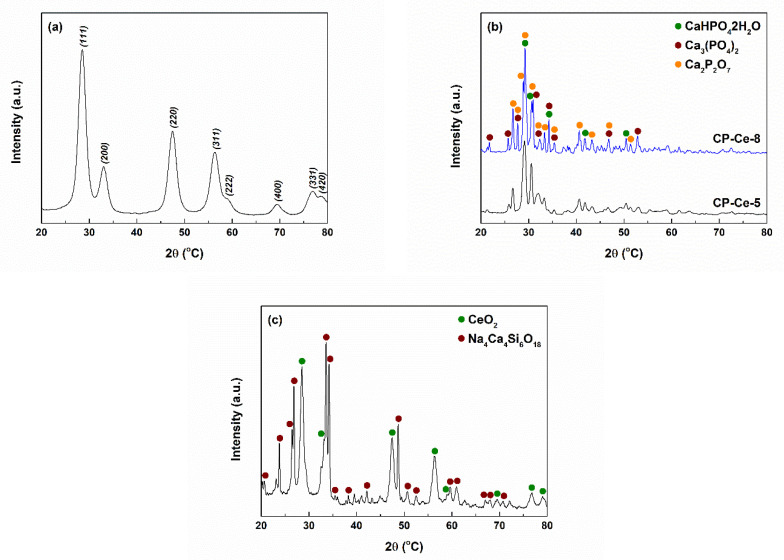
XRD patterns of the calcined powders: (**a**) CeO_2_, (**b**) BG-Ce and (**c**) CP-Ce.

According to the XRD pattern from Figure 6c, the CeO_2_ phase crystallized within the glassy matrix. CeO_2_, as an *n*-type semiconductor, has a bandgap slightly above 3 eV, depending on the processing method and morphology. Therefore, the emission band centred at about 403 nm is most likely generated by the transfer of electrons from the Ce 4f level to the O 2p level [52]. These results suggest that CeO_2_ domains that are embedded in the bioglass phase have a higher quality in terms of defect concentration.

Figure 6 depicts the XRD patterns of the calcined powders to determine the degree of crystallinity and the crystalline phases. In Figure 6a, all diffraction peaks belong to the same ordered compound, namely CeO_2_ with cubic structure (ICDD 00-081-0792). Analysing the peaks’ width, it can be stated that this material has a lower crystallinity degree, based on crystallites with small sizes (estimated at approximately 4.6 nm).

Although a ratio between Ca and P specific to hydroxyapatite was used for the synthesis, the XRD diffractograms from Figure 6b reveal the formation of a mixture of calcium phosphates. Both at 500 and 800 °C, brushite (CaHPO_4_·2H_2_O) with monoclinic symmetry (ICDD 00-072-1240), whitlockite (Ca_3_(PO_4_)_2_) with rhombohedral structure (ICDD 00-070-2065) and calcium pyrophosphate (Ca2P_2_O_7_) with tetragonal symmetry (ICDD 00-081-2257) were identified. After the treatment performed at 500 °C, brushite seems to be the major crystalline phase, followed by calcium pyrophosphate, while the temperature of 800 °C favoured the appearance of whitlockite. No distinct CeO_2_ phase was detected, which sustains the idea of Ce integration as a Ce^4+^ ion into the crystal lattice of calcium phosphates.

In the XRD pattern related to the BG-Ce powder, available in Figure 6c, the presence of two types of crystals distributed in the glassy matrix can be observed: combeite (Na_4_Ca_4_Si_6_O_18_) with hexagonal structure (ICDD 00-079-1089) and the same cubic CeO_2_ phase. The latter is more crystalline than in the previous case, with larger crystallites (estimated at approximately 10.2 nm), due to the processing at a higher temperature. However, the other ordered component (sodium calcium silicate) has an even higher degree of crystallinity, an aspect confirmed by the sharper and narrower diffraction peaks. In this way, the bioglass was converted into a bioglass–ceramic made up of a continuous vitreous matrix and two discontinuous crystalline components.

### 2.2. Scaffold Characterization

**Figure 7:**
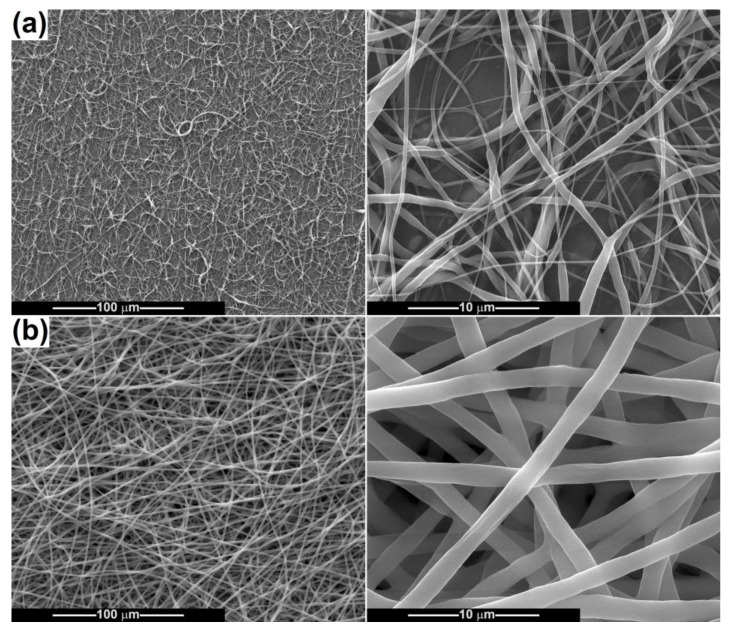
SEM images of PCL fibres: (**a**) PCL-10 and (**b**) PCL-15.

**Figure 8:**
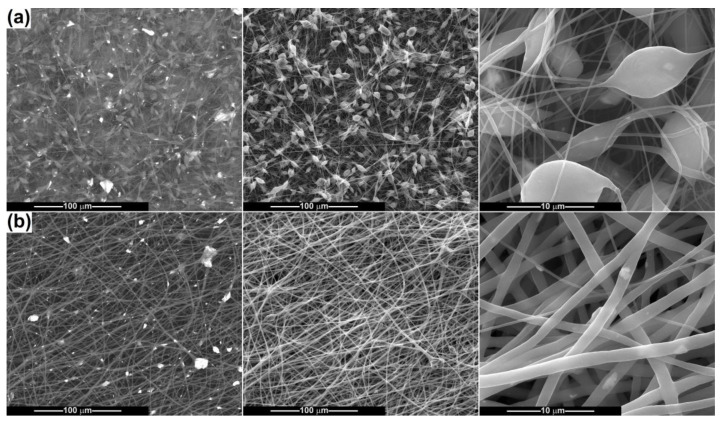
SEM images of the composite fibres: (**a**) PCL-10-CeO_2_ and (**b**) PCL-15-CeO_2_. First column represents SEM images based on backscattered electrons; the second and third columns represent SEM images based on secondary electrons, at different magnifications.

**Figure 9:**
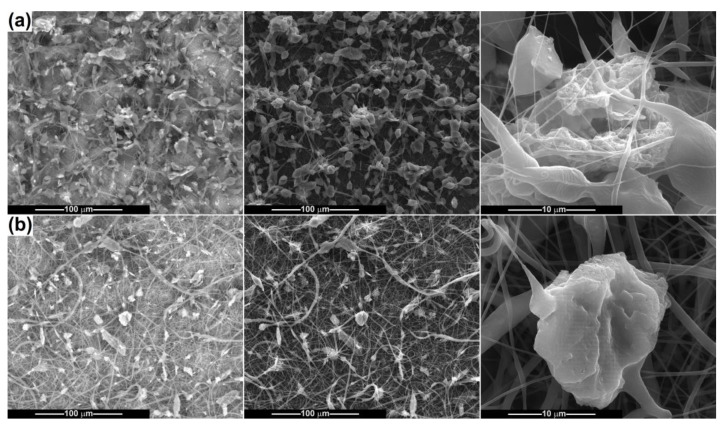
SEM images of the composite fibres: (**a**) PCL-10-CP-Ce-5 and (**b**) PCL-15-CP-Ce-5. First column represents SEM images based on backscattered electrons; the second and third columns represent SEM images based on secondary electrons, at different magnifications.

**Figure 10:**
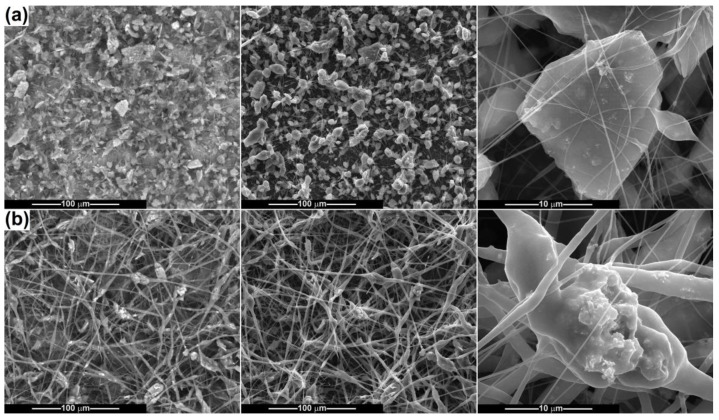
SEM images of the composite fibres: (**a**) PCL-10-CP-Ce-8 and (**b**) PCL-15-CP-Ce-8. First column represents SEM images based on backscattered electrons; the second and third columns represent SEM images based on secondary electrons, at different magnifications.

**Figure 11:**
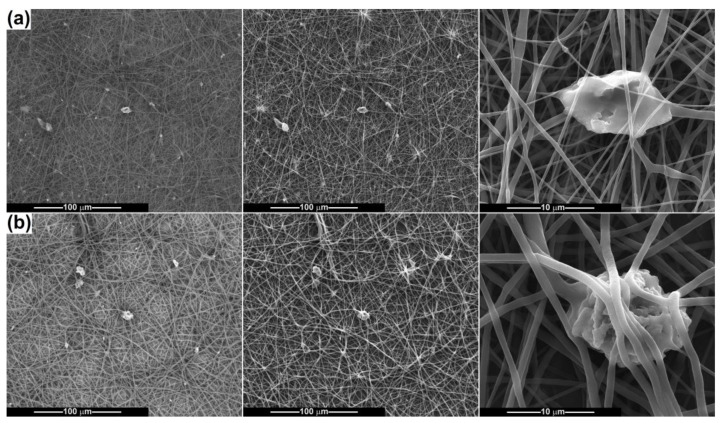
SEM images of the composite fibres: (**a**) PCL-10-BG-Ce and (**b**) PCL-15-BG-Ce. First column represents SEM images based on backscattered electrons; the second and third columns represent SEM images based on secondary electrons, at different magnifications.

After electrospinning the powder-containing polymeric suspensions, fibrous scaffolds were achieved and subsequently characterized from a morphological point of view (Figure 7, Figure 8, Figure 9, Figure 10 and Figure 11). The SEM images of PCL fibres obtained from precursor solutions of different concentrations (10 and 15 wt%) evidenced that a lower concentration leads to at least two families of fibres, some very thin, with an average diameter of 150 nm, and some thicker, with an average diameter of 700 nm (Figure 7a). The higher concentration enables the formation of coarser fibres, but more homogeneous in size, with an average diameter of 1.5 μm (Figure 7b). All of them show a smooth surface, are arranged randomly on the substrate (aluminium foil) and have a non-woven nature and a tendency to agglomerate, even to stick. In the case of 15 wt% PCL the fibres are just slightly sinuous, while for 10 wt% PCL they are quite entangled, as a proof of the instability that occurred during fibre stretching and drying. Overall, the concentration of 15 wt% is more appropriate for electrospinning high-quality fibres of constant thickness along the entire length.

By adding CeO_2_ powder in the precursor suspensions, different types of morphologies were attained, with modifications at the level of both shape and size, as can be seen in Figure 8. Because of the ellipsoidal beads that arise, the fibres from the PCL-10-CeO_2_ sample no longer have a perfect cylindrical aspect, and their diameter is variable in a wide range. In the thickened areas, the diameter increases up to 10 μm, but most of the basic fibres in Figure 8a maintain their diameter around 250 nm. To assess the degree of loading with mineral powder, as well as its spatial distribution, SEM images based on backscattered electrons were also recorded, in which the bright areas are assigned to heavier elements, Ce in this situation. Thus, CeO_2_ particles are visible as clusters of different dimensions (sometimes reaching 20 μm), embedded either in the beads’ volume or inside the fibres. Figure 8b highlights the influence of polymer concentration increasing, which triggers a dimensional homogenization of the fibres (diameter around 1.3 μm) and a more regular arrangement of the particles within them compared to the former situation. For the PCL-15-CeO_2_ sample, the powder layout seems to be more balanced and the agglomerates smaller in size.

Figure 9 and Figure 10 present the results obtained when electrospinning the suspensions containing Ce-doped calcium phosphates. As a general observation, it can be stated that the fibres’ fabrication is affected to a larger extent by the presence of such powders, which hinder the equilibrium between the surface tension and repulsion force, as well as the thinning process of the polymeric jet. When the CP-Ce powders are integrated into the 10 wt% PCL solution, the final scaffolds acquire the features of a mixture of fibres and polymeric *3D* entities, which keep a fusiform design in most cases but also adopt irregular shapes, especially in the connection points (Figure 9a). The ratio of disordered micrometric structures is even higher for the sample derived from the suspension with CP-Ce-8 (Figure 10a), maybe due to the lower efficiency of breaking up particle agglomerates during ultrasonication. However, both materials contain a spiderweb of thin fibres (150 nm average diameter) that ensures the assembly between the coarse entities and the integrity of the composite scaffold.

A concentration of 15 wt% PCL led to distinguishable modifications in morphology: the thinner fibres sometimes increase their average diameter (200–300 nm), the polymeric *3D* entities almost disappear, and instead of them thicker fibres or fibre sections occur, reaching a maximum of 5 μm. Comparing the PCL-15-CP-Ce-5 sample (Figure 9b) with the PCL-15-CP-Ce-8 sample (Figure 10b), the second is a little more homogenous from a dimensional point of view, probably because the surface energy of the particles is lower after calcination at 800 °C, and this condition affects the electrospinning process less.

According to the SEM images capable of differentiating between different atomic numbers, the agglomerates of particles are unevenly distributed and placed mostly inside the fibres, as well as partially embedded or attached to their surface in some cases.

The third situation is presented in Figure 11, namely the combination of PCL in different proportions with a bioglass–ceramic powder. This powder consists of large angular blocks that can settle at the bottom of the syringe and, therefore, the fibre loading degree is reduced, as the backscattered-electron-based SEM images emphasize. Figure 1a shows a small number of polymeric defects and fibres with thickness in a wide range (200–800 nm) for the PCL-10-BG-Ce sample, while Figure 1b evidences a smooth surface and a narrow fibre size distribution (900 nm average diameter) for the PCL-15-BG-Ce sample. This information is not surprising considering that a higher concentration of PCL ensures a better control of fibre diameter (Figure 7) and provides more material, so that thicker structures can be fabricated.

**Figure 12:**
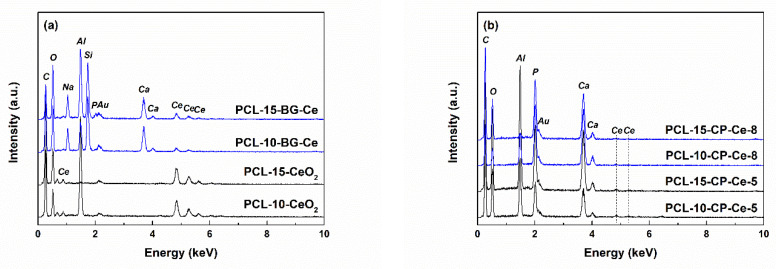
EDX spectra of the composite fibres: (**a**) PCL-CeO_2_, PCL-BG-Ce and (**b**) PCL-CP-Ce.

The presence of Ce in the final scaffolds was confirmed through the corresponding EDX spectra (Figure 12). The tall spectral lines assigned to PCL (C and O) and collector foil (Al) are accompanied by several well-defined peaks typical of Ce in the case of the CeO_2_-containing samples and multiple maxima of variable intensity specific to Si, P, Na and Ce for the samples with BG-Ce loading (Figure 12a). As it was expected, the intensity of Ce-related peaks decreases from PCL-CeO_2_ to PCL-BG-Ce, since in the second case Ce is just a dopant at a proportion of 5 mol%. The height of the maxima attributed to Ce reduces further in the EDX spectra of the PCL-CP-Ce scaffolds (Figure 12b), which is understandable if the integration of the rare earth in the crystal structure of calcium phosphates happened, leading to a balanced distribution of Ce^4+^ ions in the entire mass, as in the other two situations, when Ce is concentrated in a CeO_2_ continuous phase or isolated domains. Au was sometimes identified as part of the elemental composition due to the surface metallization to achieve conductivity.

### 2.3. In Vitro Studies

**Figure 13:**
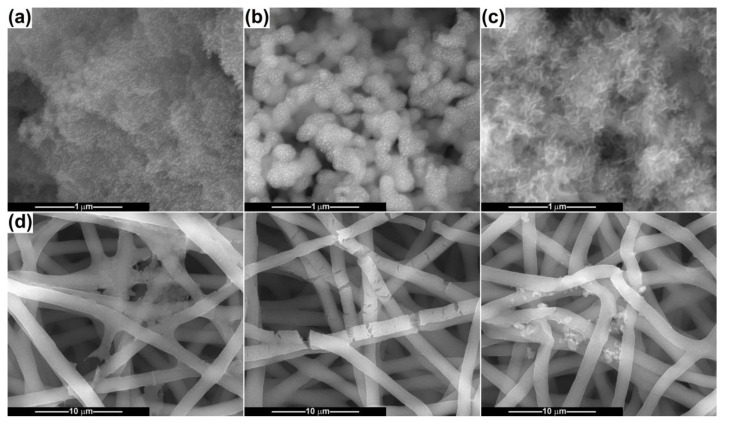
SEM images of (**a**) CP-Ce-5, (**b**) CP-Ce-8 and (**c**) BG-Ce powders and (**d**) PCL-15 fibres after immersion in SBF for 28 days.

In this study, both the bioactive powders and bare bioresorbable fibres were subjected to a long-term immersion in SBF to elucidate the repercussions of such treatment, similar to the conditions in a living organism on the morphological properties and mineralization process. The changes were investigated with the help of the SEM images centralized in Figure 13. The CP-Ce-5 and CP-Ce-8 powders display a reduced coverage with apatite after SBF immersion for 28 days, visible in the form of small brighter areas in Figure 13a,b, in correlation with their high degree of crystallinity. However, the bioglass–ceramic, which supposes the existence of a glassy phase prone to chemical attack, is completely coated with a thick new layer, which hides the substrate characteristics and looks like a crowding of fluffy clouds composed of needle or foil-type structures; this morphology corresponds to apatite, as it has been reported several times in the scientific literature [55]. This indicates that the analysed material is highly bioactive.

Regarding the PCL fibres electrospun from the solution with 15 wt% concentration, the corresponding images from Figure 13d indicate the initiation of the degradation process. Thus, sometimes the shape of the fibres becomes flattened, and other time cracks and breaks appeared. These modifications, which occurred after such a short term, validate the possibility of accelerated degradation in the SBF environment, especially if the scaffolds are maintained for a longer period. The degradation of fibres also suggests a weakening of their internal structure, which may reduce the mechanical strength of the scaffolds. The presence of cracks and discontinuities allows the SBF solution to penetrate inside the fibres, thus hastening the degradation process by providing a higher surface area or enabling the mineralization process by exposing the embedded bioactive phases.

**Figure 14:**
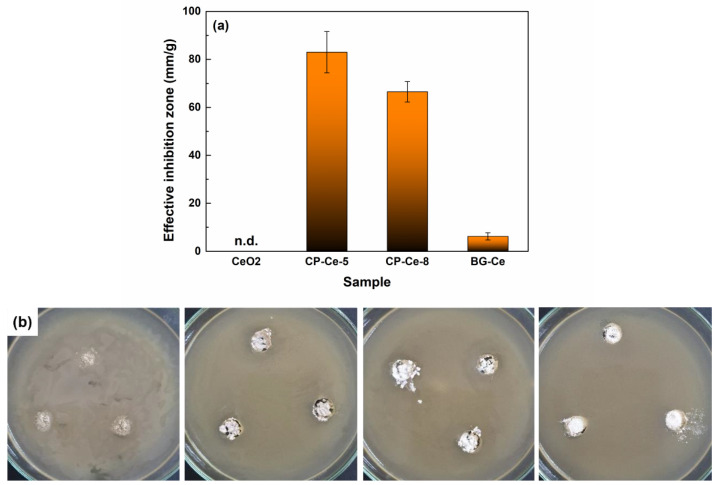
(**a**) Antibacterial activity of the powders against *E. coli* and (**b**) corresponding digital images.

The results of the antibacterial test against *E. coli*, performed on all calcined powders (Figure 14), validated the suitability of such materials as antimicrobial agents for tissue engineering. The antibiogram of the mineral powders demonstrates that these show good antimicrobial activity against colonization and biofilm development, with the CP-Ce-5 sample having the most pronounced antibacterial activity, followed by CP-Ce-8 and BG-Ce samples; since the CeO_2_ powder was not in sufficient quantity, the inhibition zone could not be determined (Figure 14a). The digital images show the bacterial biofilm development capacity on the analysed materials (Figure 14b). These findings can be explained based on Ce incorporation within the developed materials, either as a CeO_2_ crystalline phase or as a Ce^4+^ dopant ion, occupying the sites of Ca in the crystal lattice of calcium phosphates. Most likely, the switch between Ce^4+^ and Ce ^3+^, the transition that is the essential part of the antibacterial mechanism and reactive oxygen species generation, happens more easily when Ce is individually localized, not as long-range ordered domains [56].

**Figure 15:**
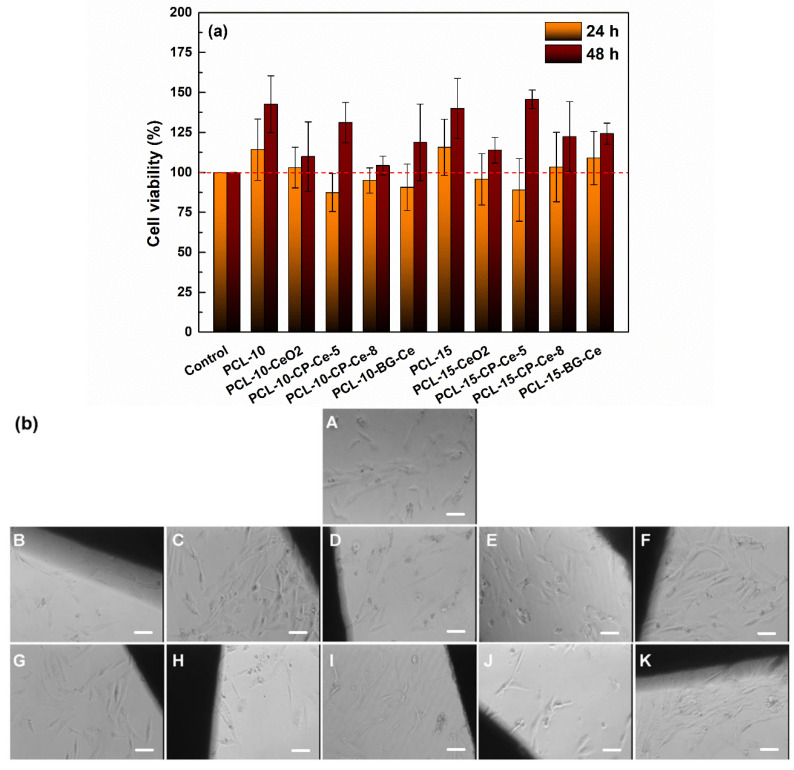
(**a**) MTT assay and (**b**) optical microscopy images of hFOB cells in contact with (A) control, (B) PCL-10, (C) PCL-10-CeO_2_, (D) PCL-10-CP-Ce-5, (E) PCL-10-CP-Ce-8, (F) PCL-10-BG-Ce, (G) PCL-15, (H) PCL-15-CeO_2_, (I) PCL-15-CP-Ce-5, (J) PCL-15-CP-Ce-8 and (K) PCL-15-BG-Ce. The images were acquired with 10× objective. The scale bar is 10 μm for all images.

To investigate the effect of the electrospun composite scaffolds containing 5 wt% mineral powder on human osteoblast cells, specific cellular tests were performed and are illustrated in Figure 15. This approach was chosen because there is information in the scientific literature indicating that an excessive amount of oxide powder can have a toxic effect on cells. To assess cell viability, the MTT assay was used, as can be seen in Figure 15a. In the case of the cells grown on the investigated materials for 24 h, the cell viability is greater than 80%, indicating that the specimens are biocompatible. When the cells were allowed to grow for 48 h, an increase in cell viability can be observed compared to 24 h and the control condition. Surprisingly, the results obtained at 48 h indicate that the prepared scaffolds favour the development of osteoblasts, with the viability reaching values between 130 and 140% for the samples PCL-10, PCL-15, PCL-10-CP-Ce-5 and PCL-15-CP-Ce-5. Similar results were obtained for MG-63 cells cultured for 7 days on bioglasses containing Ce [57].

The utility of Ce-doped materials promoting bone regeneration was reported in several studies using various cell lines. Ce-doped bioceramics reduced oxidative stress and promoted the viability of MG-63 cells [58]. Morais et al. [59] reported that Ce-doped glass has good biocompatibility, as well as cell attachment, adhesion and spreading of MG-63 cells following 4 and 7 days of growing. Other studies also confirmed the efficiency of the mesoporous bioactive glasses (MBGs) containing different Ce percentages, which showed good fibroblast (L929) cytocompatibility at both 48 and 96 h, promoting wound healing [42,55]. A recent study by Varini et al. [60] indicated that MBGs have good biocompatibility for a mouse calvaria preosteoblastic cell line (MC3T3-E1) grown for various numbers of days. P_2_O_5_-free Ce-containing glasses exhibited good bioactivity and compatibility for murine long bone osteocytes (MLO-Y4) grown for either 24 or 72 h [61].

Figure 15b presents the sample–cell interface images provided by optical microscopy following 24 h of cell growing. For all experimental conditions, it can be observed that cells retain a fusiform, bipolar shape, with an elongated cell body, comparable to the morphology of control cells (A). The results indicate that the medium coming into contact with the samples does not contain toxic components, allowing the cells found around the tested materials to attach, develop and, based also on the MTT assay, survive. It is obvious that the CP-Ce-5 powder generated the best biological properties in terms of both antibacterial activity and biocompatibility. A similar result was shown in a previous study, in which the optical images recorded for Schwan and osteoblast cells grown on Y- and Ce-containing disks evidenced that the disks support attachment and growth for the osteoblasts, as opposed to Schwan cells [62].

## 3. Materials and Methods

### 3.1. Materials

The purpose of this work resides in the obtaining of composite scaffolds based on mineral powders and a bioresorbable polymer. To attain it, the following reagents were employed: ammonium cerium(IV) nitrate ((NH_4_)_2_Ce(NO_3_)_6_, ≥98%, Sigma-Aldrich, Burlington, MA, USA), calcium nitrate tetrahydrate (Ca(NO_3_)_2_·4H_2_O, 99–102%, Merck, Darmstadt, Germany), diammonium hydrogen phosphate ((NH_4_)_2_HPO_4_, ≥99%, Merck, Darmstadt, Germany), ammonium hydroxide (NH_4_OH, 25% NH_3_, Sigma-Aldrich, Burlington, MA, USA), tetraethyl orthosilicate (Si(OC_2_H_5_)_4_, TEOS, 98%, Aldrich, Burlington, MA, USA), triethyl phosphate ((C_2_H_5_O)_3_PO, TEP, 99%, Merck, Darmstadt, Germany), sodium nitrite (NaNO_2_, 99%, Riedel-de Haën, Charlotte, NC, USA), nitric acid (HNO_3_, ≥65%, Fluka, Charlotte, NC, USA), polycaprolactone ((C_6_H_10_O_2_)_n_, PCL, *Mw* = 80,000 g/mol, Sigma-Aldich, Burlington, MA, USA), chloroform (CHCl_3_, CF, ≥99%, Sigma-Aldich, Burlington, MA, USA) and N,N-dimethylformamide (C_3_H_7_NO, DMF, 99.8%, Sigma-Aldich, Sigma-Aldich, Burlington, MA, USA).

### 3.2. Powder Synthesis

In the first part of this study, three types of Ce-containing powders were synthesized by wet-chemistry methods, namely precipitation/coprecipitation and sol-gel. Both techniques ensure superior properties in term of purity, homogeneity, particle size control and low-temperature processing.

#### 3.2.1. CeO_2_ Synthesis

To obtain 2 g of CeO_2_ powders, the necessary amount of Ce precursor (NH_4_)_2_Ce(NO_3_)_6_) was solubilized in distilled water by magnetic stirring and then the solution pH was adjusted to 10–11 by adding NH_4_OH. The initial intense orange solution turned into a yellowish suspension after precipitation. The solid was filtered, washed several times with distilled water and dried at 60 °C for 48 h. The final powder was obtained after calcination at 500 °C for 2 h (***coded CeO_2_***).

#### 3.2.2. Calcium Phosphate (CP) Synthesis

The second powder was designed starting from the formula of hydroxyapatite (Ca_10_(PO_4_)_6_(OH)_2_, HA), doped with 5 mol% Ce (Ca_9.5_Ce_5_(PO_4_)_6_(OH)_2_). To obtain 10 g of Ce-doped powder, two solutions were prepared: the corresponding quantities of Ca and Ce precursors (Ca(NO_3_)_2_·4H_2_O and (NH_4_)_2_Ce(NO_3_)_6_) were dissolved in distilled water, while P precursor (NH_4_)_2_HPO_4_) was dissolved separately in distilled water, both by magnetic stirring. The solutions were then mixed, maintaining the pH at 10–11 with NH_4_OH. The resultant precipitate of white colour was filtered, washed thoroughly with distilled water and dried at 60 °C for 48 h. The final powders were achieved after calcination at 500 °C (***coded CP-Ce-5***) and 800 °C (***coded CP-Ce-8***) for 2 h.

#### 3.2.3. Bioglass (BG) Synthesis

The third powder was processed by the sol-gel route, as opposed the first two, which were prepared by precipitation and coprecipitation, respectively. Thus, starting from the oxide composition of S53P4 bioglass, known as BonAlive^®^ commercial products, 5 mol% of Na_2_O was replaced with CeO_2_, leading to 53% SiO_2_–4% P_2_O_5_–20% CaO–18% Na_2_O–5% CeO_2_, that, in addition to bioactivity, should also exhibit pronounced antibacterial properties. After performing the calculations for 10 g of bioglass, Si and P precursors (TEOS and TEP) were hydrolysed under magnetic stirring, the first one in an acidic environment provided by HNO_3_. In addition, Ca, Na and Ce precursors (Ca(NO_3_)_2_·4H_2_O, NaNO_2_ and (NH_4_)_2_Ce(NO_3_)_6_) were solubilized in distilled water by ultrasonication. The first two solutions were added over the third one, with the final mixture being homogenized for 1 h by magnetic stirring. Next, it was placed in an oven at 60 °C for 48 h, during which gelation and maturation of the resulting gel took place, as well as its drying. The resulted material was mortared and calcined at 800 °C for 2 h (***coded BG-Ce***).

### 3.3. Scaffold Fabrication

The previously described powders were further integrated in PCL fibres, which were fabricated by electrospinning. At the beginning, a solvent mixture was obtained by mixing CF and DMF in a volumetric ratio of 4:1. Then, 5 wt% powder (CeO_2_, CP-Ce and BG-Ce) was added, and the resulting suspensions were ultrasonicated for 15 min. Finally, PCL was added in a concentration of 10 and 15 wt% and dissolved by magnetic stirring for 24 h. The electrospinning process took place in an isolated chamber to be able to control the environmental parameters, namely temperature and humidity. Thus, the parameters were set to the following values and maintained for all experiments: 0.5 mL/h feeding rate, 15 kV applied voltage, 15 cm spinneret-collector distance, 20 min duration, 22–23 °C temperature and 45% humidity. The bare and powder-containing samples were coded according to Table 1.

### 3.4. Physicochemical Characterization

Thermal analysis was performed from room temperature to 900 °C, with a heating rate of 10 °C/min in air using a Netzsch STA 449 F3 Jupiter equipment (Netzsch Group, Selb, Germany). Attenuated Total Reflectance Fourier Transform Infrared (ATR-FTIR) spectroscopy was carried out in the wavenumber range of 400–4000 cm^−1^, with 4 cm^−1^ resolution and 32 scans/sample, employing a Thermo Scientific Nicolet iS50 spectrophotometer (Thermo Fisher Scientific, Waltham, MA, USA). The morphology of the gold-coated samples was investigated by scanning electron microscopy (SEM) with a FEI Quanta Inspect F50 microscope (FEI Company, Hillsboro, OR, USA) operated at 20–30 kV accelerating voltage, 3.5 spot size and 10 mm working distance and equipped with an energy-dispersive X-ray (EDX) spectroscopy probe. Photoluminescence (PL) spectra were recorded in the wavelength range of 350–550 nm using a FL 920 Edinburgh Instruments spectrophotometer (Edinburgh Instruments, Livingstone, UK) equipped with a Xe900 lamp. X-ray diffraction (XRD) was conducted in the 2*θ* range of 20–80° with a 2°/min scan speed and 0.02° step size, employing a Shimadzu XRD 6000 diffractometer (Shimadzu Corporation, Kyoto, Japan) with Ni-filtered Cu K*α* radiation (*λ* = 0.154 nm). The average crystallite size (*D*) was estimated with the Debye–Scherrer equation:

*D* = *K*·*λ*/(*β*·cos *θ*),

where *K* is a dimensionless shape factor with a typical value of about 0.9 for spherical shape, *λ* is the X-ray wavelength (0.154 nm), *β* is the full width at half maximum (*FWHM*) value and *θ* is the Bragg angle.

### 3.5. In Vitro Studies

To study the impact of immersion in simulated body fluid (SBF), small pieces of samples were fully submerged in a solution prepared according to Kokubo et al. [63] and incubated at 37 °C for 28 days. Afterwards, the samples were extracted, rinsed thoroughly with distilled water and dried in mild conditions. The surface morphology was analysed with SEM after gold coating.

The antibacterial activity was evaluated against a Gram-negative strain, *Escherichia coli* (*E. coli*), in Nutrient Agar culture medium (Carl Roth, Karlsruhe, Germany). The pH was adjusted to neutral, while sterilization was performed at 120 °C for 20 min. The culture medium was distributed in Petri dishes, which were subsequently inoculated using the depletion technique with 100 μL fresh *E. coli* bacterial inoculum (grown for 24 h), having an optical density of 0.667, measured at 600 nm, which corresponds to the McFarland standard of 4, namely an approximate cell density of 1.2 × 10^9^ CFU/mL (*CFU*—colony forming units). The method of powder diffusion in the culture medium was applied, with 3 wells of 6 mm diameter made in the inoculated dishes and weighted amounts of sample introduced into each well. The Petri dishes were incubated at 37 °C for 24 h, then photographed and measured. The effective inhibition zone (*IZ_eff_*) values were calculated by referring to the sample mass. The experimental determinations for the antibacterial activity were performed in triplicate and the graphic representation includes the statistical standard deviation against the mean value.

For the biocompatibility assessment, hFOB 1.19 cells (human osteoblasts) were grown in an incubator with 5% CO_2_ in a humid environment. The culture medium employed for cell growth was DMEM/F12 (Dulbecco’s Modified Eagle Medium/Nutrient Mixture F-12) supplemented with 0.3 mg/mL G418 (Geneticin), 10% FBS (Foetal Bovine Serum) and 1% P/S (Penicillin/Streptomycin). The samples were cut into squares with a size of 1 cm^2^ and UV sterilized for 15 min on both sides. Next, they were placed in 24-well plates. The cells were detached from the culture flask and seeded on the investigated materials at a density of 20,000 cells/surface. These were then allowed to grow for 24 and 48 h. The same number of cells was seeded directly in the plate, representing the negative control condition. After reaching the deadline, the cells were investigated to determine if the studied scaffolds were biocompatible for osteoblasts.

Cell viability was evaluated using the MTT (3-(4,5-dimethylthiazol-2-yl)-2,5-diphenyltetrazolium bromide) assay. Metabolically active cells are able, based on NAD(P)H-dependent oxidoreductant enzymes, to reduce the tetrazolium (MTT) molecule, which presents a yellow colour, to an insoluble form of formazan, which has a purple colour. The protocol used to determine cell viability is described below. At the right time, the culture medium was removed and replaced with a solution of 1 mg/mL MTT prepared in the culture medium and left to incubate with the cells for another 4 h. Next, the medium was removed, and the insoluble formazan crystals were dissolved in dimethylsulfoxide (DMSO). Finally, the absorbance of the formazan solution was measured at 590 nm using a Mithras LB 970 plate reader (Berthold Technologies, Bad Wildbad, Germany). The obtained values were used to determine the percentage of viable cells according to the following formula:

*Viability* [%] = 100 × *A_sample_*/*A_control_*,

where *A_sample_* is the measured absorbance value for the tested material and *A_control_* is the measured absorbance value for the negative control.

Indirect effects of the disks on cells grown in the wells were imaged at 24 h following incubation using an Olympus Optical Microscope equipped with a 10× objective and CCD camera.

## 4. Conclusions

By employing the electrospinning technique, fibres based on polycaprolactone (PCL) and cerium (Ce)-containing powders were successfully fabricated. This approach allowed the incorporation of the mineral particles into the polymeric scaffold, thus providing additional properties or improving the existing ones. The powders were synthesized by two wet-chemistry methods, precipitation/coprecipitation and sol-gel. The CeO_2_ powder presented a cubic structure and nanometric morphology; calcium phosphate (CP) powders doped with Ce were also in the nanometric range, but from a compositional point of view they consisted of a mixture of different calcium phosphates in the structure of which Ce was integrated as a dopant. The bioglass powder appeared in the form of micrometric particles in which a vitreous phase and at least two crystalline phases (combeite and CeO_2_) coexist, leading to a bioglass–ceramic. The composite materials displayed a fibrous morphology, with the fibres having a smooth surface and different diameters, both on the nanometric and micrometric scale. More than that, especially for the scaffolds derived from the suspensions with a lower concentration of PCL, fusiform or irregular beads occurred on the fibres, which validates the conclusion that the fibres’ shape and size are influenced by the precursor suspension composition used in the manufacturing process. Moreover, the powders’ distribution within the final scaffolds was evaluated with the help of SEM images obtained by the detection of backscattered electrons; these showed a less homogeneous dispersion on large areas, with agglomerates of particles of a broad size distribution, depending on powder type and polymer proportion.

The powders’ bioactivity and polymer biodegradability were demonstrated through the SBF immersion test, observing a significant deposition of apatite on the bioglass and advanced fragmentation and degradation of the polymeric fibres’ surface after exposure to a simulated physiological environment for 28 days. The studies carried out on the prepared mineral powders revealed that the best antibacterial behaviour was achieved for calcium phosphates doped with Ce. To assess their applicability in the field of bone regeneration, the bare and composite scaffolds were assessed in the presence of human osteoblast cells, and the results confirmed that the proposed materials are well tolerated by cells and exhibit good cell proliferation.

Considering both the biodegradability and biocompatibility properties, as well as the antimicrobial capabilities provided by the integration of the mineral component, it can be stated that the developed composite scaffolds are materials with potential in the field of tissue engineering.

## Figures and Tables

**Table 1:** Sample codification and precursor suspension composition.

No.	Sample Code	Precursor Suspension Composition					
		PCL (g)	CF:DMF 4:1 (mL)	CeO_2_ (g)	CP-Ce-500 (g)	CP-Ce-800 (g)	BG-Ce (g)
1	***PCL-10***	1.0	10	-	-	-	-
2	***PCL-10-CeO_2_***			0.5	-	-	-
3	***PCL-10 CP-Ce-5***			-	0.5	-	-
4	***PCL-10 CP-Ce-8***			-	-	0.5	-
5	***PCL-10-BG-Ce***			-	-	-	0.5
6	***PCL-15***	1.5		-	-	-	-
7	***PCL-15-CeO_2_***			0.5	-	-	-
8	***PCL-15 CP-Ce-5***			-	0.5	-	-
	***PCL-15 CP-Ce-8***			-	-	0.5	-
	***PCL-15-BG-Ce***			-	-	-	0.5

## Data Availability

Data is contained within the article.
